# Cardiac Hepatopathy: New Perspectives on Old Problems through a Prism of Endogenous Metabolic Regulations by Hepatokines

**DOI:** 10.3390/antiox12020516

**Published:** 2023-02-17

**Authors:** Alexander A. Berezin, Zeljko Obradovic, Tetiana A. Berezina, Elke Boxhammer, Michael Lichtenauer, Alexander E. Berezin

**Affiliations:** 1 Internal Medicine Department, Zaporozhye Medical Academy of Postgraduate Education, 69000 Zaporozhye, Ukraine; 2 Klinik Barmelweid, Department of Psychosomatic Medicine and Psychotherapy, 5017 Barmelweid, Switzerland; 3 Department of Internal Medicine & Nephrology, VitaCenter, 69000 Zaporozhye, Ukraine; 4 Department of Internal Medicine II, Division of Cardiology, Paracelsus Medical University Salzburg, 5020 Salzburg, Austria; 5 Internal Medicine Department, Zaporozhye State Medical University, 69035 Zaporozhye, Ukraine

**Keywords:** heart failure, cardiac hepatopathy, inflammation, oxidative stress, hepatokines, adropin, fetuin-A, alpha-1-microglobulin, fibroblast growth factor-21, selenoprotein P

## Abstract

Cardiac hepatopathy refers to acute or chronic liver damage caused by cardiac dysfunction in the absence of any other possible causative reasons of liver injury. There is a large number of evidence of the fact that cardiac hepatopathy is associated with poor clinical outcomes in patients with acute or actually decompensated heart failure (HF). However, the currently dominated pathophysiological background does not explain a role of metabolic regulative proteins secreted by hepatocytes in progression of HF, including adverse cardiac remodeling, kidney injury, skeletal muscle dysfunction, osteopenia, sarcopenia and cardiac cachexia. The aim of this narrative review was to accumulate knowledge of hepatokines (adropin; fetuin-A, selenoprotein P, fibroblast growth factor-21, and alpha-1-microglobulin) as adaptive regulators of metabolic homeostasis in patients with HF. It is suggested that hepatokines play a crucial, causative role in inter-organ interactions and mediate tissue protective effects counteracting oxidative stress, inflammation, mitochondrial dysfunction, apoptosis and necrosis. The discriminative potencies of hepatokines for HF and damage of target organs in patients with known HF is under on-going scientific discussion and requires more investigations in the future.

## 1. Introduction

The number of new cases of heart failure (HF) is steadily increasing worldwide. The prevalence of HF with preserved ejection fraction (HFpEF) is currently higher than that of HF with reduced (HFrEF) and mildly reduced (HFmrEF) ejection fraction [1,2,3]. The exact absolute risks for HF progression from stage A to stage C have remained stable over the past decade (8.4 per 100 person-years), regardless of the implementation of conventional strategies [4]. Previous observational studies found that one-year mortality in HF varied widely from 4% to 45% depending on the presence of acute or chronic HF, HF phenotype, age, gender and concomitant diseases [4,5,6]. According to the Acute Heart Failure Database (AHEAD) registry, liver function test abnormalities (elevations in total bilirubin, γ-glutamyltransferase, alkaline phosphatase, aspartate aminotransferase and alanine aminotransferase) were found in 76% of patients with known acute HF [7]. The PROTECT trial (Placebo-controlled Randomized study of the selective A1 adenosine receptor antagonist KW-3902 for patients hospitalized with acute HF and volume Overload to assess Treatment Effect on Congestion and renal funcTion) and the ASCEND-HF trial (Acute Study of Clinical Effectiveness of Nesiritide in Decompensated Heart Failure) showed that acute HF including cardiogenic shock was a meaningful cause of cardiac liver dysfunction [8,9]. Moreover, patients with acute or actually decompensated HF were significantly more likely to have abnormalities in liver test than patients with chronic HF [9]. On the other hand, new-onset liver injury due to cardiac dysfunction has been shown to be a strong predictor for HF progression, higher risks of hospitalization and poor clinical outcomes (all-cause mortality and cardiovascular death) and quality of life [9,10,11]. Along with it, there is evidence of the fact that a severity of liver injury secondary to progressive HF was loosely associated with adverse cardiac remodeling, skeletal muscle dysfunction and sarcopenia/cardiac cachexia [12,13].

However, in HF, multiple pathophysiological interactions between neurohumoral and systemic/local inflammatory activations, as well as an altered immune response, lead not only to adverse cardiac remodeling but also to worsening hepatic circulation and sequelae following the development of acute cardiogenic liver injury and congestive hepatopathy [14,15,16]. The pathogenesis of cardiac hepatopathy is complex and involves underlying canonical molecular mechanisms that overlap with the development and progression of HF (i.e., centrilubular liver necrosis, dilated sinusoids and perisinusoidal fibrosis due to hypoperfusion associated with fluid retention and passive congestion, altered electrolyte and protein metabolism, iron homeostasis and secondary portal hypertension). Additionally, other pathophysiological pathways link liver dysfunction to renal dysfunction, altered endothelial function, anabolic/catabolic imbalance, abnormalities in the intestinal microbiome, impaired metabolism of adipose tissue and skeletal muscle [17,18,19]. Less is known about the role of hepatokines (adropin, fetuin-A, selenoprotein P, alpha-1-microglobulin) produced by hepatocytes as a result of oxidative stress and microvascular inflammation in progressive dysfunction of other organs in HF, including adverse cardiac remodeling, renal damage, adipose tissue inflammation and skeletal muscle myopathy [20]. The majority of hepatokines are synthesized and released mainly by hepatocytes (adropin, fibroblast growth factor-21 [FGF-21], alpha-1-microglobulin), whereas others (fetuin-A and fetuin-B) are additionally produced by various tissues including adipose tissues. They exerted their modality to regulate liver metabolism and energy homeostasis, local synthesis and releasing inflammatory cytokines, activation of stellate cells and fibroblasts, local antigen-presenting cells, and provided immune adaptive, anti-inflammatory, anti-oxidative, anti-apoptotic and protective effects with remote organs (heart, lung, kidney, pancreas) and tissues (vasculature, white adipose tissue, pericardial adipose tissue, skeletal muscles) [21]. Although all these processes are crucial for HF progression, because they affect target organ dysfunction, vascular integrity and tissue reparation, understanding underlying molecular mechanisms in connection with their regulation by hepatokines retains uncertain. On the other hand, it remains unclear whether hepatokines cause the direct and initial attack on the heart inducing cardiac remodeling through activating Akt, nuclear factor-κB (NF-κB), and ERK signaling and/or chances to get damaged due to other organs damage-associated chemical mediators like acute renal failure and skeletal muscle dysfunction or, in contrast, their act as adaptive modulator of endogenous repair system [22]. The aim of this study was to increase the knowledge of hepatokines as adaptive regulators of metabolic homeostasis in patients with HF.

## 2. Definition, Morphological Criteria, and Biochemical Profiling of Liver Damage in HF

The current paradigm of cardiac hepatopathy refers to any hepatocyte injury caused by acute or chronic cardiac dysfunction in the absence of clear evidence of other possible causes of liver injury [21,22]. Although there is no consensus on the definition and terminology of cardiac hepatopathy, the majority of experts use the terms congestive cardiac hepatopathy (CCH) and acute cardiogenic liver injury (ACLI) to describe two different aspects of the disease [23,24,25,26]. CCH is the most common disease in HF caused by passive venous congestion of the liver. Several chronic cardiac diseases, such as constrictive pericarditis, tricuspid regurgitation, primary and secondary cardiomyopathies, inherited cardiac defects and cardiac hypertrophy, associated with chronic HF, usually lead to congestion and thus to the development of CCH [27,28]. There is ample clinical evidence that CCH is closely related to HFrEF/HFmrEF and corresponds to the New York Heart Association (NYHA) HF functional class [29]. In contrast, ACLI is most commonly associated with arterial hypoperfusion and downstream hypoxia due to acute HF resulting from acute myocardial infarction, acute decompensated chronic HF, progressive natural history of severe myocarditis and cardiomyopathies [30]. Nevertheless, the role of primary hypotension in the development of ACLI is controversial. For example, a retrospective analysis of a cohort of 31 HF patients with clinical and biochemical evidence of ACLI revealed that hypotension alone cannot be considered as a trigger of acute liver injury [31]. Rather, other factors such as multiple concomitant diseases in collaboration with various epigenetic influences are implicated for the occurrence of ACLI [32].

Histologically, these two phenotypes of cardiac hepatopathy appear sufficiently distinct. In most cases, CCH is characterized by sinusoidal dilatation associated with extravasation of red blood cells from sinusoids into periportal areas and replacement of hepatocytes by red blood cells, as well as the expression of necrotic areas without severe cellular infiltration and apoptosis of cells in the third zone of Rappaport acini [21,33]. To note, there is evidence of the fact that the small diameter of fenestrae in sinusoids in humans is likely to be a serious obstacle for hepatocyte transduction, so that higher variability of clinical presentation of CCH due to passive congestion may relate to genetic reasons [33]. The extension of periportal necrotic zones together with secondary centrilobular necrosis and accumulation of fibrosis tissue may eventually lead to ineffective intrahepatic circulation supporting ischemia/hypoxia and loss of functional hepatocytes, endothelialization of sinusoids and development of liver cirrhosis in advanced cases [21,34]. The main histological findings in ACLI are massive necrosis of the third zone of Rappaport acini, gross deformation of the liver parenchyma, large fibrotic areas along with rapidly progressive portal hypertension and splenic hypertrophy, often leading to the early onset of hepato-renal syndrome [21,35].

In this context, the primary laboratory findings of cardiac hepatopathy vary depending on numerous factors that include not only the phenotype of the disease, but also the duration of hypotension, the type of cardiac dysfunction (left-sided, right-sided, or biventricular), the presence of comorbidities, patient age and gender [36,37]. Indeed, the greatest increase in transaminases was observed in patients with right-sided or biventricular HF than in left-sided HF [37]. Meanwhile, the prevalence of CCD in HF patients depended on a signature of comorbidity, male gender and older age [7,8,9]. Thus, cardiac hepatopathy may manifest as asymptomatic elevation of serum levels of aminotransferases and/or bilirubin and severe liver injury with significant elevation of aminotransferases, alkaline phosphatase, γ-glutamyltranspeptidase, lactate dehydrogenase and a decrease in plasma albumin levels. Clinical signs of cholestasis, ascites, peripheral edema, portal hypertension and concomitant oligoanuria may not be closely related to the phenotype of cardiac hepatopathy [38]. However, regardless of its phenotype, cardiac hepatopathy has strong prognostic value in identifying all-cause mortality, cardiovascular events and mortality associated with HF and has some particular implications for the management of patients undergoing cardiac assist device implantation or heart transplantation [39].

## 3. Common Underlying Molecular Mechanisms of Cardiac Hepatopathy

**Figure 1:**
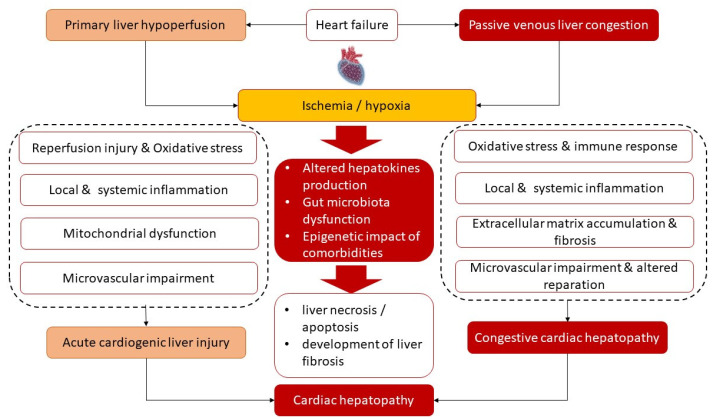
Most common underlying mechanisms of the pathogenesis of cardiac hepatopathy.

Figure 1 illustrates the main underlying molecular mechanisms of the development of both phenotypes of cardiac hepatopathy. Primary liver ischemia/reperfusion and/or passive liver congestion with secondary tissue ischemia/hypoxia together with oxidative stress are thought to trigger a secondary locally and systemically inflammatory cascade that induces microvascular impairment, intrahepatic thrombosis, liver necrosis/apoptosis, and development of liver fibrosis with its conversion to cirrhosis and interorgan interactions during HF pathogenesis. Along with it, there is assumption of a causative role of sinusoidal thrombi, which are direct reason for tissue ischemia and fibrosis. In addition, alterations in liver function lead to progressive changes in splanchnic blood flow, coronary circulation and systemic hemodynamics and thereby maintain liver fibrosis progression. Finally, cardiac hepatopathy is supported by inadequate secretion of various hepatokines, such as adropin, fetuin-A, FGF-21, alpha-1-microglobulin and selenoprotein P, which influence energy metabolism, local and systemic inflammation and immune response.

### 3.1. Ischemia/Reperfusion and Inflammation/Fibrosis Cascade

Ischemic injury of the liver is a component of ACLI induced by hypoxia due to hypoperfusion and is characterized by sequential damage to intracellular organelles and whole cells, cell swelling and further persistent disruption of Na+/K+-ATPase function [40,41]. The intracellular accumulation of low-oxidized lipids and proteins, overexpression of xanthine oxidase and NADPH oxidase leads to overproduction of reactive oxygen (ROS) and nitrogen species (such as peroxynitrite, hypochlorite), contributing to acidosis-induced suppression of mitochondrial transmembrane malate-aspartate exchange and carnitine-related mechanism of acyl-CoA transfer, and inducing ischemic mitochondrial dysfunction leading to alteration of mitochondrial permeability and ATP depletion [42]. In fact, the additional damage to liver tissue is a consequence of the paradoxically exacerbating restoration of perfusion by oxygen/Ca2+ delivery [43]. Finally, ROS, intracellular calcium overload, inflammatory cytokines (interleukin [IL]-1b, IL-2, tumor necrosis factor-alpha [TNF]) and chemokines (hypoxia-inducible factor-1α, C-X-C motif ligand-8, C-C motif ligand-2, C-C motif ligand-10) support the early activation of Kupffer cells, the late activation of polymorphic mononuclear cells and the accumulation of CD4+ T lymphocytes. Additionally, these processes activate stellate cells, mononuclear cells and platelets in perisinusoidal spaces and periportal areas and the post-ischemic disruption of liver microcirculation together with a decrease in sinusoidal density in liver parenchyma [44,45]. Antigen-presenting cells and CD4+ T lymphocytes secrete a variety of growth factors such as TNF-β, granulocyte-macrophage colony-stimulating factor and interferon gamma, which enable direct activation of Kupffer cells and promote their ability to synthesize and release inflammatory cytokines [46]. Meanwhile, in ACLI, nitric oxide levels were found to be sufficiently reduced [47]. Moreover, an imbalance between nitric oxide production by nitric oxide synthase and endothelin-1 leads to vasoconstriction of sinusoids and exacerbates the vicious cycle of altered blood circulation [48]. Overall, ischemic and post-ischemic oxidative stress and mitochondrial injury are considered potent triggers for further overexpression of inflammatory genes and activation of hepatocellular apoptosis, ferroptosis and necrosis during hepatic ischemia/reperfusion injury in acute HF [49]. Indeed, Tanaka et al. (2014) [49] found that numerous apoptotic hepatocytes in the third zone of Rappaport acinar were co-localized with NADPH oxidase 4 (NOX4) and that these findings were a consequence of hypoxia-induced intrahepatic microcirculatory failure but were not induced by activated APCs, such as macrophages and mononuclear cells. On the other hand, hepatocyte energy metabolism, as measured by determining the local activities of citrate synthase, carnitine palmitoyltransferase-1 and cytochrome c oxidase, was increased by inflammatory cytokines despite ultrastructural changes in mitochondria during hepatocyte apoptosis [50]. It is possible that the transmission of death signals in the early phase of hepatocyte apoptosis is mainly associated with the co-stimulation of APCs, while in the late phase it is regulated by other signals such as NOX4 and active caspase-3, Bax and Bcl-2 that might be regulated via Toll-like receptor-4 (TLR4)/phosphatidylinositol 3-kinase (PI3K)/protein kinase B (Akt)/glycogen synthase kinase 3-beta (GSK-3β) signaling [51]. However, local production of inflammatory cytokines mediates expression of cell surface adhesion molecules (intracellular cell adhesion molecule, vascular cell adhesion molecule) on the surfaces of hepatocytes and endothelial cells, induces adherence of APCs and consequently leads to intravascular/intra-sinusoidal coagulation, which contributes to microcirculatory failure and autophagy [52,53]. Interestingly, ischemia/reperfusion-induced liver injury may be promoted by M1 polarization of macrophages via regulation of peroxisome proliferator-activated receptor-γ (PPAR-γ) in response to increasing anaerobic glycolysis and accumulation of lactic acid in the microenvironment [54]. Evidence suggests that polarization of hepatic M1 macrophages, which supports acute and chronic liver injury, can be promoted by gut-derived exosomes following intestinal ischemia/reperfusion [53,54]. Thus, decreased splanchnic blood flow is an independent factor contributing to liver injury. However, all these triggers prolong hepatic ischemia/hypoxia and exacerbate apoptosis/necrosis, creating a vicious cycle of excessive inflammatory response involving activated antigen presenting cells with excessive cytokine and ROS production, leading to further oxidative liver tissue damage [55,56].

The molecular pathways contributing to CCH are not different from those mentioned above, while ischemia is secondary to passive liver congestion and the reperfusion phase is closely related to decompensation of HF associated with unstable hemodynamics. Indeed, CCH is not as dramatic compared with ACLI, so the histological sequelae are not necessarily the same as those of ACLI. Initial dilatation of hepatic sinusoids due to passive liver congestion is associated with a degree of venous pressure in vena porta and results in the exudation of red blood cells, activated mononuclear cells, platelets and protein rich-fluid into the perisinusoidal space of Disse. However, low-grade inflammation and excessive fibrosis play a more significant role in the pathogenesis of CCH than in ACLI. There is strong evidence that inflammatory chemokine signaling (C-X-C motif ligand-8, C-C motif ligand-2, C-C motif ligand-10) derived from activated Kupffer cells induces polarization of monocyte-derived macrophages into the M1 phenotype and stimulates fibrogenesis through activation of hepatic stellate cells [57,58,59]. Another difference between CCH and ACLI concerns the advocacy of hepatic angiogenesis [60]. Indeed, hypoxia-induced vascular endothelial growth factor (VEGF) expression has been found to be crucial in advanced fibrosis, whereas inflammation in early stages of fibrotic transformation of the liver may involve VEGF in hepatic angiogenesis [60,61]. However, the low regenerative capacity of liver progenitor cells seems to be closely related to altered mitophagy, which is a selective form of autophagy for damaged and/or redundant mitochondria [62,63]. Indeed, pro-inflammatory cytokines such as TNF-alpha perform variable cellular responses, including cell proliferation, metabolic activation, inflammatory responses and apoptosis, acting through the PI3K/Akt pathway, transforming growth factor (TGF)-β signaling, Fas aggregation and NF-κB signaling [64,65,66]. There is strong evidence that TGF-β and pro-inflammatory cytokines synergistically promote collagen synthesis and support fibrosis via the pSmad3C pathway [66,67]. Last but not least, ferroptosis seems to be more sufficient than apoptosis for chronic ischemia-induced CCH [68]. Ferroptosis is triggered by nuclear factor E2-related factor 2, but not by hypoxia-inducible factor-1α, which is often considered to trigger apoptosis in ACLI [68,69]. In summary, CCH is considered to be a mild-to-moderate progressive disease that shows histological signs of progression from liver fibrosis to liver cirrhosis at the advanced stage of its natural evolution.

### 3.2. Intestinal Microbiota

Recent data show that the secretome of the gut microbiota may be involved in the regulation of liver regeneration after acute and chronic liver injury [70]. Therefore, different microbiota profiles were found to be associated with the rate of decompensation HF and thereby intervened in outcome in these patients [71]. Indeed, a wide range of secretory components, including gut-derived lipopolysaccharides, bile acids associated with gut microbiota and numerous bacterial metabolites (short-chain fatty acids and tryptophan metabolites), may influence protective capacity by protecting hepatocytes from injury and supporting repair [72]. Although there is no certain explanation for the transmission of signals from the microbiota to hepatocytes [73], the secretome of the intestinal microbiota is thought to mediate activities of the farnesoid X receptor (FXR)-fibroblast growth factor 19 (FGF19) axis and enhance the TGR5-glucagon-like peptide axis, which are involved in the metabolic regulation of proliferative response and attenuation of immunological imbalance [74,75]. In addition, lipopolysaccharide (LPS) derived from bacterial walls of the intestinal microbiota and bacterial DNAs, that are known TLR9 agonists, have been found at elevated levels in the peripheral blood of patients with various liver diseases [76,77]. Increased permeability of the intestinal vascular barrier to such macromolecules is the result of sufficient disruption of splanchnic blood flow, edema of the intestinal wall, free fluid in the peritoneum and maldigestion [78]. Finally, simultaneous stimulation of antigen-presenting cells by LPS and bacterial DNAs may lead to activation of the inducible form of nitric oxide synthase and release of nitric oxide, which is accompanied by systemic vasodilation and hyperdynamic changes in the circulation to prevent impaired perfusion of distant tissues [77,79,80]. Yet, several metabolites of the microbiota, such as trimethylamine N-oxide, tryptamine and indole-3-acetate, may attenuate inflammatory responses, insulin resistance, mitochondrial and endoplasmic reticulum dysfunction through binding with endoplasmic reticulum stress kinase PERK (EIF2AK3) and activation of transcription factor FoxO1, as well as they may mediate the expression of fatty acid synthase and sterol regulatory element-binding protein-1c in hepatocytes [81,82]. Normally, these metabolites reduced fatty-acid- and LPS-stimulated ability of macrophages to synthetize and release pro-inflammatory cytokines and inhibited the migration activity of T cells and mononuclears toward a chemokine [83]. Depletion of the microbiota in HF was associated with liver fibrosis due to LPS-stimulation of stellate cells and, however, promoting local iron sequestration through ferroportin-expressing phagocytes and hepatocyte-derived hepcidin acting as activator of conventional dendritic cells [83]. To note, about 30% of HF patients had no changes in the microbiota, while bacterial translocation from the gut microbiota and persistence of bacterial antigens were assumed to be responsible for systemic inflammation and HF decompensation. These facts indicate that the gut microbiota may influence liver architectonic and perfusion through other mechanisms, which need to be discovered [71]. For instance, there is evidence of the fact that the modifications to the microbiota by bile acid may provide signals through the intestine and bacterial products, as well as incretins and adipocytokines produced in the bowel, which affect lipid metabolism of hepatocytes [84,85]. Accumulation of triglycerides along with specific lipids including lysophosphatidylcholine and ceramides derive the toxic effects on hepatocytes and leads to mitochondrial and endoplasmic reticulum dysfunction due to oxidative stress, activation of signaling cascades and death receptors, and apoptosis [86]. Overall, the gut microbiota acts as modulator of oxidative stress and inflammatory response in liver tissue.

### 3.3. Adipose Tissue Dysfunction

Adipose tissue is involved in various changes in HF patients (inflammation, browning) and it is a source of mesenchymal stem cells and adipokines, which essentially regulate the energy metabolism of distant tissues including the liver [87,88,89]. Adipogenic liver transformation propagates local redox imbalance and activates in lipid-dependent deterioration of hepatocytes. Alterations in hepatocyte histology is associated with elevated expression of PPARγ, adipocyte protein, interleukin-6 (IL-6), interleukin-18 (IL-18), CD36 and adiponectin [90]. It appears that PPARα and PPARδ dysfunction is a key regulator, through which adipokines can mediate repair processes in the liver [91]. Nevertheless, altered autophagy/mitophagy and mitochondrial dysfunction may be attenuated by adiponectin in both ACLI and CCH [92]. It is possible that adiponectin induces autophagosome formation through AMP-activated protein kinase (AMPK)-dependent activation of Unc-51-like kinase 1, which subsequently leads to the removal of damaged mitochondria from hepatocytes [92]. Indeed, suppression of autophagy in white adipose tissues attenuated the liver fibrosis through adipose-liver crosstalk [93]. However, adiponectin protects liver injury by inactivating the TGF-beta-1/SMAD2 pathway and exerts an anti-fibrotic effect via the AMPK/STAT3 pathway [94]. However, adipokines (adiponectin, leptin) are thought to contribute to the anti-inflammatory effects of various hepatokines, such as apelin, adropin and fetuin-A by decreasing the local production of IL-6 and fibroblast growth factor 21 (FGF21), which prevent hepatic steatosis and fibrosis [95,96,97,98,99]. At the same time, apelin mediats Fas-induced liver injury in part via activation of c-Jun N-terminal kinases [100]. In parallel, FOXO transcription factors, which are directly modulated by insulin signaling in the liver, can be downregulated by IL-6, TNF-alpha and follistatin and can be upregulated by FGF21 [101]. Thus, adipokines that modulate the synthesis of IL-6, TNF-alpha, FGF21/Klotho and nitric oxide production via extracellular regulated kinase1/2 (p-ERK1/2) may improve liver metabolism by indirectly regulating glucose homeostasis and preventing mitochondrial dysfunction [102,103,104]. Meanwhile, FGF21 acts on adipocytes and renal cells to induce synthesis of angiotensin-converting enzyme 2 that inhibits hypertension and reverses vascular damage and microvascular inflammation [99]. Overall, the functional pleiotropism of adipose tissue affects its ability to synthesize and secretes numerous adipokines, which regulate liver metabolism, thermogenesis, microvascular inflammation, liver and remote tissue reparations, angiogenesis, insulin resistance, endothelial integrity, and causes not only multiple serious conditions, including adverse cardiac remodeling, adipose tissue dysfunction, liver fibrosis, but also mediates adaptive local changes in target organs.

### 3.4. Impaired Skeletal Muscles Metabolism

Skeletal muscle myopathy is a common condition of HF, especially in patients with HFrEF and HFmrEF, which is strongly associated with a decrease in effective perfusion [105]. However, skeletal muscle cells have endocrine capabilities and secrete a broad spectrum of regulatory proteins called myokines (myonectine, irisin, myoststin, FGF21, FGF15), which are involved in autocrine/paracrine regulation of distant organs and tissues, including the liver [106]. They are able to activate AMP-activated protein kinase and modulate its cooperation with the transcription factor nuclear respiratory factor 1, sirtuin-1 and the transcriptional co-activator peroxisome proliferator-activated receptor-γ co-activator 1α, and induce the expression of mitochondrial transcription factor-A genes that support mitochondrial protection [107,108,109]. Loss of a pool of these secreting proteins such as irisin, myonectin, FGF21 and growth differential factor-11 (GDF11) reduces the tissue-protective effect of myokines [110,111,112]. However, a precise understanding of the beneficial effects of myokines on cardiac hepatopathy appears to require further investigation.

### 3.5. Epigenetic Impact of Metabolic Comorbidities on Liver Tissue Modification

Epigenetic changes in DNA through DNA methylation, histone modifications and microRNA sequences are thought to be critical for specific changes in genes that coordinate several underlying molecular mechanisms in the pathogenesis of cardiac hepatopathy [113]. In addition, various metabolic comorbidities such as diabetes mellitus, insulin resistance and nonalcoholic fatty liver disease are thought to influence the reparative capacity of the liver through epigenetic changes in genes encoding adipokines, transport proteins (microsomal triglyceride transfer protein, patatin-like phospholipase domain-containing protein 3), PPAR-γ receptors and also genes involved in adipogenic/lipogenic regulation (homeostasis-associated gene, retinoid X receptor alpha gene, and liver X receptor alpha gene) [114,115,116,117]. However, epigenetic regulation of liver tissue susceptibility to acute and chronic damage in HF is the subject of further investigation.

Thus, the occurrence of cardiac hepatopathy is the result of several overlapping mechanisms, which include ischemia/reperfusion injury of liver tissue, passive fluid congestion, reduced hepatic blood with intrahepatic thrombosis, total body hypoxemia, and inability to utilize oxygen and metabolic components. These mechanisms are under close regulation of auto-paracrine and epigenetic influences. Increasing evidence suggests that interplay between hepatokines, adipokines and cardiokines mainly natriuretic peptides are crucial for the metabolic crosstalk between systemic and local myocardial/microvascular inflammation in HF and peripheral tissue damage in liver and some remote organs and tissues including kidney, spleen, skeletal muscle and adipose, tissue), but the direct underlying mechanisms linking advanced cardiac dysfunction and liver fibrosis remain not fully elucidated.

## 4. Plausible Role of Hepatokines in Cardiac Hepatopathy

### 4.1. Adropin

Adropin is a low molecular weight, multifunctional, membrane-bound peptide synthesized predominantly in the liver and brain [118]. The expression of adropin is regulated by dietary behavior and nutrients, as well as by various inflammatory cytokines and adipokines, including leptin [119,120]. There is interesting evidence of a link between the Energy Homeostasis Associated gene, which encodes adropin, energy homeostasis and lipid metabolism [119]. However, adropin mediates the expression of PPAR-γ receptor and hepatic lipogenic genes [120]. Adropin is expressed in numerous tissues, including liver, heart, brain, pancreatic tissue, kidney, small intestine, endothelial cells, colostrum and vessels [121].

Physiologically, adropin acts by binding to the orphan G protein-coupled receptor and the liver X receptor to suppress water deprivation and enhance glucose and lipid oxidation, respectively [122,123]. However, it remains unclear how adropin enhances intrahepatic metabolism and supports integrative intracellular signaling activities in the liver. One of the explanations concerns the ability of adropin to attenuate insulin-mediated regulation of glucose homeostasis and insulin resistance. Another assumption is based on the positive effect of adropin on the integrity of the endoplasmic reticulum through suppression of cAMP-activated protein kinase A and c-Jun N-terminal kinase activity in hepatocytes, resulting in decreased phosphorylation of the inositol triphosphate receptor and decreased secretion of NLRP3 inflammasome [121,122]. It is possible that all these factors reduce the efficacy of genes related to inflammation. There are numerous data on tissue-protective effects of adropin resulting from activation of the GPCR-MAPK-PDK4 pathway (protecting the heart and hepatocytes from metabolic dysfunction), VEGF receptor-2 (supporting vascular integrity, endothelial function, capillary density, and angiogenesis), PI3K-Akt and Erk1/2 pathways (promotion of nitric oxide production) and the MAPK and FOXO1 pathways (suppression of inflammation and attenuation of oxidative stress) [123,124,125,126,127,128,129].

In clinical practice, low adropin levels were detected in patients with abdominal obesity, diabetes mellitus, arterial hypertension, multifocal atherosclerosis, chronic kidney disease and chronic HF, whereas increased adropin levels have been found in acute HF and acute coronary syndrome [128,129,130,131,132]. Furthermore, circulating adropin levels were negatively correlated with cardiovascular disease risk in the general population [132,133,134,135,136]. There is a limited amount of strong evidence for a positive role of adropin in liver injury. Chen et al. (2019) [137] reported that adropin protects against acute liver injury through binding to Nrf2, mediating antioxidant capacity and attenuating hepatocyte necrosis via upregulating liver expression of Gclc, Gclm and Gpx1. Therefore, adropin has demonstrated its ability to reduce the local production of IL-6 and TNF-alpha, thereby suppressing the intensity of inflammatory response, along with attenuating mitochondrial dysfunction in hepatocytes and propagating fibrosis [125,138,139]. An experimental study by Skrzypski et al. (2022) [140] showed that exogenous adropin improved glucose control and restored the levels of elevated transaminases in peripheral blood without modulating insulin sensitivity in animals with type 2 diabetes mellitus and concomitant liver injury. However, supporting adropin synthesis in the liver is thought to be effective in liver protection. Indeed, there is evidence that activation of opioid μ-receptors and glucagon-like peptide 1 receptors in the liver can increase the circulating adropin pool, opening new perspectives for liver-protective therapy [141,142].

### 4.2. Fetuin-A

Fetuin-A (also known as alpha2-Heremans-Schmid glycoprotein) is a low molecular weight multifunctional protein synthesized predominantly by the liver [143]. Approximately 5% of the fetuin-A produced is extrahepatic localized. After extensive post-translational modifications of its single-chain precursor, Fetuin-A is recognized as a biologically stable form in the bloodstream and unfolds its biological potency [144]. Clearance of fetuin-A is provided by a widely distributed cysteine peptidase. The most important biological role of Fetuin-A is its participation in various metabolites (minerals, lipids) and binding of lectins [145]. Fetuin-A is involved in a number of pathophysiological regulations, such as insulin receptor signaling, adipocyte dysfunction and inflammation, liver fibrosis, lipid toxicity, triacylglycerol production, macrophage phenotype modification, promotion of angiogenesis, β-cell damage/apoptosis and TLR4 activation, which are critical for liver integrity and function [146,147,148]. Therefore, fetuin-A is responsible for downregulation of insulin receptor expression and TGF-beta signaling [149,150].

In the clinical setting, higher fetuin-A levels were strongly associated with insulin resistance, abdominal obesity, T2DM, extravascular calcification, asymptomatic atherosclerosis, cardiovascular disease, chronic kidney disease, nonalcoholic fatty liver disease and cognitive dysfunction [150,151,152,153,154,155]. It has been postulated that elevated fetuin-A levels may be an adaptive response to counteract the production of inflammatory cytokines [156]. In addition, there are conflicting data on an association between variability in circulating fetuin-A associated with single nucleotide polymorphisms in the fetuin-A-encoding AHSG gene and T2DM risk in the general population [157,158,159]. Whether the SNP can be associated with liver complications in HF remains uncertain. Meanwhile, circulating fetuin-A levels were sufficiently reduced (by 64%) in patients with acute liver failure compared with those who did not have liver failure [160]. Nevertheless, fetuin-A predicts progression of liver and vascular fibrosis in hemodynamically stable patients with cardiovascular disease and nonalcoholic fatty liver disease [161]. In contrast, there was no clear evidence that circulating fetuin-A levels have significant predictive value for liver fibrosis in patients without T2DM and cardiovascular disease [162]. Fetuin-A is thought to modulate an impact of dietary inflammatory potential on liver parenchymal integrity and fibrosis risk [163]. Possibly, in this way, this hapotokine attenuates a negative influence of circulating LPS/DNA from the gut microbiota on inflammatory changes in the liver by acting as a regulator of HMGB1 release from innate immune cells [164]. However, Tomita et al. (2022) [165] reported that low fetuin-A concentrations were found in the circulation of patients with HF compared with healthy subjects. Moreover, decreased fetuin-A concentrations were associated with hepatic hypoperfusion, but not with liver stiffness [165]. Finally, the authors found that HF patients with low fetuin-A concentrations and liver hypoperfusion had the lowest survival rate. Potentially, fetuin-A could be a new surrogate biomarker for cardiac hepatopathy with possible discriminatory value.

### 4.3. Alpha1-Microglobulin

Alpha1-microglobulin is a small, multifunctional circulating glycoprotein that belongs to the lipocalin family and is synthesized exclusively by the liver and abundantly released into the bloodstream [166,167]. Its major biological role involves endogenous protection against a broad spectrum of oxidants, including inhibition of proteinases, methemoglobin, cytochrome C, oxidized collagen I, oxidized low-density lipoprotein, free iron and mediation of tissue repair [168]. Alpha1-microglobulin acts as stimulator for APCs through activating Akt, NF-κB, and ERK signaling systems and, consequently, it enhances inflammation, migration and polarization of macrophages [23]. On the contrary, alpha1-microglobulin exerted its ability to suppress fibrogenesis-related mRNA expression in cultured macrophages and cardiac fibroblasts [23]. Intramyocardial AM administration in animals with acute myocardial infarction activated migration of macrophage, their infiltration of myocardial damage area, inflammatory response, and enhance matrix metalloproteinase-9 mRNA expression in the infarct area and peri-infarct zones, whereas disturbed fibrotic repair, then provoked acute cardiac rupture in acute myocardial infarction [23]. Alpha1-microglobulin is encoded by the alpha1-microglobulin bikunin precursor gene (AMBP), which also encodes bikunin, a structural protein of the extracellular matrix and a Kunitz-type plasma proteinase inhibitor [169]. Under physiological conditions, AMBP gene expression in the liver is regulated by hepatocyte nuclear factors and the Keap1/Nrf2 system. AMBP mRNA has also been detected in numerous tissues other than the liver, including the placenta, retina, cardiac muscle, skeletal muscle, kidney, lung and vasculature. Up-regulation of the AMBP gene has been found in numerous diseases characterized by increased ROS levels and circulating proteases [170,171,172]. AMBP functions as an intravascular and extravascular radical scavenger with high reduction activity and the ability to bind free heme groups, as well as chaperones [173,174]. In fact, AMBP downregulates the expression of apoptotic, inflammatory and stress-related genes, improves membrane permeability, prevents mitochondrial damage, and reduces renal and liver tissue damage from acute ischemia/hypoxia-induced injury [174,175,176]. Although AMBP is a liver-derived glycoprotein exclusively, its clinical significance has been better explored in acute kidney injury and acute nephrotoxicity than in ACLI [177,178]. However, the predictive value of AMBP in cardiac hepatopathy seems promising and requires further investigation.

### 4.4. Fibroblast Growth Factor-21

Fibroblast growth factor-21 (FGF21) is a stress-induced peptide produced mainly by the liver, but also by adipose tissue and skeletal muscle [179]. In the liver, it acts as a Klotho/β-Klotho cofactor and regulates mitochondrial oxidative activity and gluconeogenesis, increasing insulin sensitivity, decreasing plasma glucose levels, and modulating lipolytic activity [180]. In white adipose tissue (WAT), FGF21 regulates WAT browning along with brown adipocyte activation and lipolysis [180]. Moreover, FGF21 increases the expression of uncoupling protein 1 (UCP1) and other thermogenic genes, thus stimulating the expression of adiponectin in WAT [181]. Administration of FGF21 shows a wide range of beneficial responses in animals with obesity-related metabolic disorders, including reduction of fat mass, alleviation of postprandial hyperglycemia, attenuation of insulin resistance, improvement of dyslipidemia, hepatic autophagy and prevention of steatohepatitis via histone demethylase Jumonji-D3 (JMJD3/KDM6B) [182,183]. Moreover, FGF21 deficiency promotes the occurrence of steatosis, inflammation, oxidative stress, autophagy, hepatocyte damage and excessive fibrosis in the liver [184]. Although protective effects of FGF21 have been attributed to the support of endothelial integrity and function, attenuation of lipid accumulation and atherosclerotic plaque formation and the inhibition of cardiomyocyte apoptosis and regulation of the oxidative stress-inflammation cascade [185], the protective mechanisms for the liver have not been fully elucidated. Indeed, FGF21 has demonstrated its ability to remarkably decrease the levels of circulating transaminases, IL-6 and TNF-alpha [186]. Notably, there is evidence for the involvement of SIRT1 autophagy signaling in protective and antifibrotic effects of FGF21 in response to acute liver injury [187,188]. Besides, FGF21 seems to have the ability to suppress ferroptosis and consequently to induce significant protection of hepatocytes from iron overload-induced mitochondrial damage leading to necrosis and excessive fibrosis [189]. However, FGF21 can regulate PGC-1α expression in hepatocytes and attenuate apoptosis and fibrosis-related gene expression in the liver [190]. The role of FGF21 interactions with the central nervous system in the context of liver injury is currently under scientific debate [191]. It is possible that FGF21 could link altered eating behavior to cardiac hepatopathy in HF patients with cardiac cachexia, as beta-adrenergic stimulation of thermogenic gene expression requires FGF21 [192]. Last but not least, FGF21 mediated the activation of ERK1/ERK2 in WAT, liver and skeletal muscle, mediating the systemic protective effects on energy metabolism by improving insulin sensitivity [193]. The clinical significance of these findings needs to be investigated in detail in the future.

### 4.5. Selenoprotein P

Selenoprotein P is a liver-derived secretory redox protein with an intrinsic thioredoxin domain whose major biological function is the transfer of selenium to intracellular glutathione peroxidases [194]. Selenoprotein P is considered as a hepatokine with antioxidant capabilities that prevents mitochondrial dysfunction and oxidative stress in numerous tissues and organs including the liver [195,196]. Therefore, in the physiological state, selenoprotein P regulates systemic energy metabolism and pancreatic β-cell function, and prevents its apoptosis by suppressing caspase-3 activity [197]. In the pathological state, low levels of selenoprotein P were associated with impaired insulin sensitivity, altered angiogenesis and cell proliferation through inhibition of vascular endothelial growth factor [198,199]. The systemic tissue-protective effects of selenoprotein P are promoted by phosphoinositide 3-kinase/Akt and Erk 1/2 signaling pathways [200].

In ACLI, selenoprotein P is involved in pre- and post-treatment [201]. Circulating levels of selenoprotein P show a tendency to decrease in patients with acute and chronic liver disease, including acute liver injury, simple steatosis and nonalcoholic liver disease [190]. However, selenoprotein P levels were significantly increased in patients with HF with liver hypoperfusion compared with patients without this disease [202]. Interestingly, there were no significant differences between selenoprotein P levels in HF patients with and without liver congestion [203]. Selenoprotein P supplementation in patients with HF and ACLI is being actively studied. The majority of patients involved in the studies had acute alcoholic access [204,205]. Of note, no significant differences in liver structure measured by magnetic resonance imaging were found in patients with different metabolic diseases depending on their selenoprotein P status [206]. Finally, the clinical outlook for selenoprotein P seems promising, whereas there are limited data for its role in cardiac hepatopathy.

Thus, hepatokines exert local hepatic effects and promote effects on remote tissues including myocardium, vessels, skeletal muscles, and pancreas (Table 1). Although hepatokines play a crucial role as metabolic regulator of liver function and other organs in HF, their diagnostic and predictive abilities for HF require deep investigation with subsequent validation and comparison with conventional circulating biomarkers of liver injury and cardiac remodeling. Therefore, there is no sufficient data of plausible difference of sensitivity and specificity of these biomarkers in different phenotypes of cardiac hepatopathy.

More investigations in the future are needed to clearly elucidate whether hepatokines are promising molecules with predictive potency for patients with HF and cardiac hepatopathy.

### 4.6. Hepatokines and Liver Drug Metabolism

Cardio-hepatic interactions in HF implicated in liver drug metabolism and mediated toxicity impact of the agents on several tissues and organs [207]. Abnormalities in drug metabolism may affect intrinsic activity of metabolic enzymes including methylation, oxidation, hydroxylation, conjugation, reductions in the synthesis of transporters, bioavailability of active metabolites of some drugs in intestine and their hepatic clearance, and binding with plasma proteins [208]. Although there is clear the Food and Drug Administration and the European Medicines Evaluation Agency guidelines for the administration of several pharmacotherapies in renal dysfunction, specific recommendations regarding personally adjusted medication dose in patients with cardiac hepatopathy are not updated regularly [209]. To note, many potential drug interactions and co-administered agents may suppress hepatic metabolism and maintain liver injury in either hemodynamically stable or are unstable patients with acute**/**chronic HF [30]. In this context, the utilization of hepatokines reflecting of hepatocyte metabolism appears to be promising. For instance, animal study has shown that fetuin-A predicted laboratory signs of hepatotoxicity and the liver tissue infiltration of monocytes in acetaminophen-induced liver injury [209]. Less known about an influence of HF guideline-recommended treatment on hepatokines. SGLT2 inhibitors along with antagonists of renin-angiotensin-aldosterone antagonists and beta-blockers demonstrated their ability to increase in adropin levels [210]. In fact, the levels of hepatokines may be modulated by conventional HF therapy, but there is not fully understood, whether it would be powerful tool for ongoing HF drug toxicity monitoring and surrogate indicator of early response on the treatment with further changes in drug administration regime to prevent drug toxicity.

## 5. Conclusions

Hepatokines may play a crucial pathogenetic role in onset and progression of cardiac hepatopathy. They are considered novel diagnostic and predictive markers for both phenotypes of cardiac hepatopathy. The most clinically relevant hepatokines linking clinical outcomes of HF and liver structure abnormalities are adropin in ACLI and fetuin-A and FGF21 in CCH. On the contrary, FGF21 and selenoprotein P appear to be the most promising therapeutic targets among the other hepatokines in patients with any forms of cardiac hepatopathy.

## Figures and Tables

**Table 1:** Local and systemic effects of hepatokines involved in the pathogenesis of cardiac hepatopathy.

Hepatokines	Local Liver Effect	Systemic Effects	References
**Adropin**	↓ activation of hepatic stellate cells, ↓NLRP3 inflammasome, ↓ inflammatory gene expression, ↓ oxidative stress, ↓ lipid peroxidation, ↓ lipid toxicity, ↓ liver injury and fibrosis, ↓ autophagy, ↑ glucose metabolism, ↑ insulin sensitivity, ↑ anti-apoptotic and antioxidant effects, ↑ pre-conditioning	↓ adverse cardiac remodeling, ↓ systemic inflammatory reaction, ↑ vascular integrity/endothelial function, ↑ renal and splanchnic blood flow, ↑ angiogenesis, ↑ NO production	[118,122,125,126,138,139,140,141,142]
**Fetuin-A**	↓ liver inflammation, injury and fibrosis, ↓ necrosis and apoptosis, ↓ fasting glucose levels, ↑ mitochondrial function, ↑ lipid metabolism, ↑ angiogenesis	↑ antioxidant capacity, ↑ endothelial function, ↓ WAT dysfunction, ↓ inflammation, ↓ pancreatic beta-cell damage/apoptosis, ↓ adverse cardiac remodeling, ↓ vessel calcification, ↑ skeletal muscle energy metabolism, ↑ NO production	[144,145,146,147,148,149,150,151,152,153,154,155,156,157,158,159,160,161,162,163,164,165]
**Alpha1-microglobulin**	Anti-oxidative effects, ↓ apoptosis, ↓ mitochondrial damage, ↓ autophagy, ↓ hepatocyte damage, ↓ activation of hepatic stellate cells,	↓ cardiac, lung and kidney injury, ↑ anti-ischemic protection	[170,171,172,173,174,175,176,177,178]
**Fibroblast growth factor-21**	↓ mitochondrial oxidative stress, ↓ autophagy, ↓ fasting glucose levels, ↑ gluconeogenesis, ↑ insulin sensitivity	↓ WAT inflammation, ↓ lipolysis in WAT, ↓ fibrosis in myocardium, ↑ endothelial function, ↓ microvascular inflammation, ↑ NO production	[179,180,181,182,183,184,185,186,187,188,189,190,191,192,193]
**Selenoprotein P**	↓ oxidative stress, ↓ lipid peroxidation, ↓ lipid toxicity, ↓ liver injury and fibrosis	↓ pancreatic beta-cell apoptosis, ↑ antioxidant capacity, ↑ angiogenesis, ↑ insulin sensitivity	[194,195,196,197,198,199,200,201,202,203,204,205,206]

Notes: ↓, decrease; ↑, increase. Abbreviations: WAT, white adipose tissue; NO, nitric oxide.

## Data Availability

Not applicable.
